# Maternal postpartum morbidity in Marrakech: what women feel what doctors diagnose?

**DOI:** 10.1186/1471-2393-13-225

**Published:** 2013-12-05

**Authors:** Bouchra Assarag, Dominique Dubourg, Abderrahmane Maaroufi, Bruno Dujardin, Vincent De Brouwere

**Affiliations:** 1 National Institute of Health Administration, BP: 6329 Rabat, Morocco; 2 Institute of Tropical Medicine, Nationalestraat 155, B-2000 Antwerp, Belgium; 3 Université Libre de Bruxelles, route de Lennik 808, 1070 Brussels, Belgium

**Keywords:** Maternal health, Postpartum maternal morbidity, Women’s perceptions, Morocco

## Abstract

**Background:**

Information about postpartum maternal morbidity in developing countries is limited and often based on information obtained from hospitals. As a result, the reports do not usually reflect the true magnitude of obstetric complications and poor management at delivery. In Morocco, little is known about obstetric maternal morbidity. Our aim was to measure and identify the causes of postpartum morbidity 6 weeks after delivery and to compare women’s perception of their health during this period to their medical diagnoses.

**Methods:**

We did a cross-sectional study of all women, independent of place of delivery, in Al Massira district, Marrakech, from December 2010 to March 2012. All women were clinically examined 6 to 8 weeks postpartum for delivery-related morbidities. We coupled a clinical examination with a questionnaire and laboratory tests (hemoglobin).

**Results:**

During postpartum consultation, 44% of women expressed at least one complaint. Complaints related to mental health were most often reported (10%), followed by genital infections (8%). Only 9% of women sought treatment for their symptoms before the postpartum visit. Women who were aged ≥30 years, employed, belonged to highest socioeconomic class, and had obstetric complications during birth or delivered in a private facility or at home were more likely to report a complaint. Overall, 60% of women received a medical diagnosis related to their complaint, most of which were related to gynecological problems (22%), followed by laboratory-confirmed anemia (19%). Problems related to mental health represented only 5% of the diagnoses. The comparative analysis between perceived and diagnosed morbidity highlighted discrepancies between complaints that women expressed during their postpartum consultation and those they received from a physician.

**Conclusions:**

A better understanding of postpartum complaints is one of the *de facto* essential elements to ensuring quality of care for women. Sensitizing and training clinicians in mental health services is important to respond to women’s needs and improve the quality of maternal care.

## Background

In light of trends in the worldwide maternal mortality, it is commonly acknowledged that the fifth Millennium Development Goal is not likely to be achieved by 2015 [1]. Nevertheless, maternal mortality represents only the tip of the iceberg of the much larger issue of maternal morbidity [2]. Estimates suggest that among the 136 million women who give birth every year, about 1.4 million experience life-threatening medical emergencies, 9.5 million experience other complications, and 20 million have long-term disabilities [3-6]. These morbidities have different etiologies; some are related to the quality of care during pregnancy and delivery, and others are related to a broader set of social and personal factors.

Depending on the definition, we have different estimates for maternal morbidity. If we take severe acute maternal morbidity as defined by the World Health Organization (WHO) in 2010 as “a woman who nearly died but survived a complication during pregnancy, childbirth, or within 42 days of pregnancy termination through care in health facilities,” [7], prevalence rates of life-threatening medical emergencies in many countries varies from 0.6% to 14.98% using disease-specific criteria, 0.04% to 4.54% using management-based criteria, and 0.14% to 0.92% using organ-based dysfunction based on Mantel criteria [8]. Rates are higher in the low- and middle-income countries of Asia and Africa (eg, Benin, Cote d’Ivoire, Morocco, Indonesia) [9,10]. These variations might be due to real epidemiological differences in different populations or to differences in the definitions and approaches of maternal morbidity.

The lack of harmonization of these criteria makes it difficult to establish priorities in terms of assistance and treatment in maternal and infant health, especially in countries with limited resources. Morbidity data are vital for policymakers and health care planners who need to know how many women require essential obstetric care [11,12]. Studies on postpartum morbidity have been scarce in Morocco. Only one study, conducted in 2001, measured the proportion of hospital admissions for acute maternal morbidity during pregnancy, childbirth, and postpartum [13]. Usually, many reproductive disorders go unnoticed, because they cause no symptoms or only nonspecific symptoms. Because of a lack of or incomplete information due to poor documentation, we measured self-described postpartum morbidity in a group of postpartum women living in Marrakech, along with their sociodemographic factors, to compare women’s postpartum complaints with medical diagnoses.

## Methods

### Context

In Morocco, antenatal and postnatal care is provided in all 2,689 public health centers and in the private sector. Antenatal coverage is about 77% (92% in urban areas and 63% in rural areas). About half of pregnant women have their prenatal consultation in the private sector. Most deliveries take place in public hospitals (51.5%) or in the 606 first-level public delivery houses managed by midwives and general practitioners (12%); 26.8% of women deliver at home. Only 9.2% of women deliver in private hospitals [14].

### Study site

This descriptive study was carried out in Al Massira health district. This district, 10 km outside of Marrakech, Morocco, has a population of 106,000. Maternal health services in the district are offered by a public ‘delivery house’ (peripheral primary level), a private clinic, 16 private general physicians, three private laboratories, and 11 private pharmacies. In case of complications, women are referred to the secondary-level regional hospital or the tertiary-level university hospital in Marrakech.

### Study population

Our study included all women aged 18 to 49 in the Al Massira district who had delivered between December 2010 and March 2012 in the delivery house, hospital maternity wards, or private clinics. Women who had delivered at home were also eligible for inclusion, and we identified them through the civil registration system and during their newborns’ BCG vaccination.

### Postpartum consultation procedures

**Figure 1:**
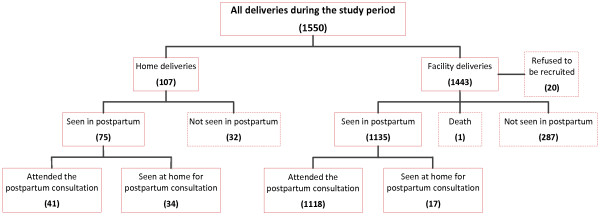
Study design.

There were 1,550 deliveries recorded in Al Massira district; 20 of these women, all single, refused to answer questions or be recruited. Thus, 1,530 women were initially included in the study and were interviewed by a midwife about their socioeconomic and demographic characteristics using a pretested questionnaire (Figure 1). The midwife invited them to attend a postpartum consultation at the nearest delivery house 6 weeks after delivery after a prepaid laboratory blood examination. The consultation consisted of a 30-minute interview followed by semistructured and open-ended questions about any postpartum complaint they had experienced and a physical examination by a doctor.

Psychological morbidity was identified through a few symptoms representing mental distress (eg, feeling negative about yourself, crying easily, decreased interest or pleasure in daily activities) in the last 2 weeks that represented a change from normal (Additional file 1 adapted from the 2001 WHO report [15]). Seven doctors and nine midwives were trained as investigators by a professor of obstetrics and gynecology from the Marrakech University Hospital. The 2-day interactive training consisted of teaching the standard operating procedure for interviewing and examining women and discussing the case definitions (Additional file 1 adapted from [16-19]).

If a woman missed her appointment, the principal investigator tried to reach her by telephone. If she then requested that the consultation be done at her home, we had a female physician fill out the questionnaire and conduct the medical examination at home. If the woman refused to participate, she was excluded from the study. All women who received a diagnosis of any morbidity or disability were treated by the study doctor or referred to a specialized hospital, where they received care free of charge, depending on their diagnosis. The conditions identified between the sixth and the eighth weeks postpartum were defined on the basis of standard clinical protocols and specific tests, as indicated in Additional file 1. These definitions were operationalized and in line with the WHO guidelines [20].

### Data analysis

Data were entered into an Epi Info™ Version 3.5.3 database. IBM SPSS Statistics for Windows, Version 20, was used for analysis. Descriptive analysis was used for sociodemographic and reproductive characteristics. To determine the socioeconomic characteristics of the women and their households, quintiles of wealth were generated using principal-component analysis based on the eligibility criteria used by Moroccan authorities to classify insurance beneficiaries (eg, possession of household commodities, homeownership). Pearson’s chi-square test was used for the crude association between the women’s postpartum morbidities and selected reproductive and sociodemographic characteristics. Women’s complaints were standardized and categorized to facilitate their comparison with diagnoses established by the medical doctor.

The standardization of complaints was performed by grouping complaints with similar symptoms into one category (ie, “constipation,” “did not go to the toilet,” “did not go to the toilet for a week” were grouped into the category “constipation”). If a woman had several complaints, we prioritized the first. For the analysis, we calculated the cumulative number of complaints for all women and then categorized them, excluding complaints without a direct link to the delivery and problems related to previous diseases (eg, asthma, goiter).

We classified the clinical diagnoses established by the medical doctors according to their definitions and clinical signs. We grouped similar diagnoses into single categories and developed a standard classification (eg, vaginitis, vulvar-vaginitis, cervicitis, vulvitis, and candidiasis were grouped under the category “genital infections”). If we had several different diagnoses for the same woman, we calculated the proportions of each of the cumulative diagnoses using the number of women consulting after delivery as the denominator.

### Ethical consideration

The study protocol was approved by the Ethical Committee of the Institute of Tropical Medicine Antwerp, the University Hospital of Antwerp in Belgium, and the Ethical Committee for Biomedical Research of Mohammed V University of Morocco in November 2010. We informed all eligible women about the purpose of the study and their rights and collected their written and oral consent before they were enrolled.

## Results

Of the 1,530 women recruited, 1,210 women attended a postpartum consultation. One death was reported following a hemorrhagic episode during delivery complicated by the Hemolysis Elevated Liver enzymes Low Platelet count (HELLP) syndrome. Regarding the sociodemographic characteristics of the 1,210 participants, 6% were younger than 20 years, and 44% were older than 30. Mean age was 29 years. Only 13 were single, and 23% had not received any formal education (Table 1).

**Table 1:** Reproductive and sociodemographic characteristics of women (N = 1,530)

**Characteristic**	**Number (%)**		***P *** **value**
	**Women with postpartum consultation**	**Women without postpartum consultation**	
	**n = 1,210**	**n = 320**	
**Age**			
< 20	68 (6)	24 (7)	0.6
20–25	271 (22)	66 (21)	
25–30	338 (28)	96 (30)	
30–35	336 (28)	83 (26)	
>35	197 (16)	50 (16)	
Unknown	0	1 (0)	
**Education level**			
None	273 (22)	118 (37)	0.0001
Primary	406 (34)	106 (3)	
High school	356 (29)	70 (22)	
Higher education	175 (15)	25 (8)	
Unknown	0 (0)	1 (0)	
**Medical insurance**			
No	892 (74)	248 (78)	0.1
Yes	318 (26)	71 (22)	
Not known	0 (0)	1 (0)	
**Occupation**			
No	1102 (91)	292 (91)	0.9
Yes	108 (9)	27 (9)	
Unknown	0	1 (0)	
**Homeowner**			
No	584 (48)	159 (50)	0.7
Yes	612 (51)	160 (50)	
Unknown	14 (1)	1 (0)	
**Socioeconomic score (quintile)**			
Poorest	189 (16)	110 (34)	<0.001
Less poor	198 (16)	0 (0)	
Average	266 (22)	133 (42)	
Less rich	239 (20)	60 (19)	
Richest	283 (23)	16 (5)	
Unknown	35 (3)	1 (0)	
**Obstetric history**			
**Parity**			
1	382 (32)	137 (43)	0.0001
2–3	672 (55)	154 (48)	
4 or more	156 (13)	28 (9)	
Unknown	0 (0)	1 (0)	
**Delivery structure**			
At home	75 (6)	32 (10)	0.0001
University hospital	78 (7)	27 (8)	
Private sector	119 (10)	14 (4)	
Regional maternity	75 (6)	35 (11)	
Delivery house	863 (71)	211 (66)	
Unknown	0 (0)	1 (0)	
**Type of delivery**			
Vaginal	576 (48)	177 (55)	0.01
Vaginal + episiotomy	426 (35)	102 (32)	
Cesarean section	122 (10)	28 (9)	
Vacuum or forceps	86 (7)	12 (4)	
Unknown	0 (0)	0 (0)	

Three hundred-twenty (21%) women did not attend the postpartum consultation. In general, these women had a lower level of education (p = 0.0001), a lower socioeconomic score (p < 0.001), and higher parity (p = 0.0001). Among the 1,210 women who attended a postpartum consultation, 893 (74%) did not have medical insurance, and 91% were unemployed. Most women (87%) had fewer than three children, and 32% were primigravidae. Nineteen percent of women had had at least one abortion (data not shown). The vast majority (94%) of women had delivered in a health facility; more than two-thirds (71%) had delivered in a delivery house, 10% in a private hospital, 7% at the university hospital, and 6% at the regional hospital. In 79% of deliveries, women decided on their own where to deliver their baby. More than one-third (35%) had at least one complication during delivery. Life-threatening emergencies represented 6% of the hospital’s deliveries in our study.

### Postpartum problems

During the postpartum consultation, 538 (44%) of the 1,210 women expressed at least one complaint, for 608 cumulative complaints (Table 2). However, 91% of women did not consult a doctor for these complaints. Of women who did not have complications during delivery, 42% mentioned a complaint that occurred during the 6- to 8-week postpartum phase. During the postpartum consultation, mental distress (anxiety, unexplained crying, nervousness) was the most commonly cited complaint (10%), followed by genital infections (vaginal discharge, vaginal leaking) (8%), and breast problems (5%). Other gynecological and obstetric problems (sexual problems, uterine prolapse, and infected episiotomy) were also reported (10%). Finally, burning during urination and urinary leakage were reported in 2% and 1% of women, respectively.

**Table 2:** Complaints by women who attended the postpartum consultation (n = 1,210)

**Cumulative complaint categories during postpartum (6–8 weeks after delivery)**	**Frequency**	%*
Psychological	124	10
Vaginal discharge	98	8
Hemorrhoids	70	6
Breast problems	63	5
Weariness	35	3
Problems of episiotomy	29	2
Prolapse	29	2
Sexual problems	27	2
Anal problems	25	2
Urinary burning	22	2
Bleeding	20	2
Vulvar itching	19	2
Pelvic problems	17	1
Hypertension	15	1
Urinary leakage	11	1
Other^1^	21	2
**Total**	608	50

*The proportion was calculated using data from the 1,210 women who had a postpartum consultation. However, a woman could have reported more than one complaint.

^1^Other: infection of cesarean section wound, cycle disorder, heart palpitations.

### Diagnoses in postpartum period

A total of 729 (60%) women received a diagnosis of a complication from a medical doctor. Among them, 9% received more than one diagnosis. The most common medical diagnosis was related to gynecological problems (22%) (genital infection, uterine prolapse, cystocele, bad repair of an episiotomy), followed by laboratory-confirmed anemia (19%). Mental distress were one of the least common diagnoses (5%). Doctors based their diagnosis of anemia on the blood cell count and classified it as mild anemia (9 < Hb ≤11 g/dL), moderate (Hb ≥7-9 g/dL), or severe (Hb < 7 g/dL). Sixteen percent of women received a diagnosis of mild anemia, 3% had moderate anemia, and one woman had severe anemia (Table 3).

**Table 3:** Cumulative number of medical diagnoses (n = 1,210)

**Cumulative category of diagnoses during postpartum (6–8 weeks after delivery)**	**Frequency**	%*
Gynecological	266	22
Anemia	224	19
Hemorrhoids	110	9
Breast	68	6
Psychological	64	5
Perianal	55	4
Hypertension	23	2
Urinary incontinence	22	2
Urinary infection	20	1
**Total**	852	71

*The proportion was calculated using data from the 1,210 women who had a postpartum consultation. However, the same woman could have had more than one diagnosis.

### Self-complaints versus medical diagnoses

Complications diagnosed by doctors did not always correspond to women’s self-reports. When we compared their self-reported complaints to the medical diagnoses made during the postpartum consultation, the most divergent proportions were mental distress and anemia (Table 4).

**Table 4:** Self-reported complaints versus observed diagnoses during postpartum consultation

**Morbidity (n = 1,210)**		**Complaints**		***P *** **value (chi square)**
		**Yes**	**No**	
**Medical diagnoses**	Yes	458 (85)	272 (40)	0.0001
n (%)	No	80 (15)	400 (60)	
**Total**		538 (100)	672 (100)	

We used bivariate analysis to assess the strength of the association between the selected demographic and reproductive characteristics and the women’s self-reports of postpartum complaints.

We found the highest prevalence of complaints reported by women ≥ 30 years, women who were employed, women who had delivered at home or in the private sector, and those who had complications during delivery (Table 5).

**Table 5:** Factors influencing women’s reported complaints in the postpartum period

**Determinants**	**Women with complaints n (%)**	***P *** **value (chi square)**
**Age**		
**<** 30 years	277 (41)	0.003
≥ 30 years	261 (49)	
**Employed**		
No	481 (44)	0.04
Yes	57 (53)	
**Place of health care delivery**		
First-level delivery house	347 (40)	0.0001
Second-level regional maternity	43 (57)	
**Place of delivery**		
Facility health	494 (44 )	0.01
Home	44 (58 )	
**Sector of delivery**		
Public sector	431 (42)	0.004
Private sector	63 (53)	
**Complications during delivery**		
No	333 (42)	0.02
Yes	205 (49)	

## Discussion

Information on postpartum morbidity in developing countries is limited and, when available, usually describes the type of medical condition diagnosed at the hospital level. This is the first study conducted in Morocco comparing postpartum morbidity diagnosed by a physician vs. self-reported morbidity among women. In this study, 44% of women reported postpartum problems. This prevalence is high, and the morbidities were a mixture of benign and severe. The most common type of problem was psychological (10%) and reveals that mental distress that should be addressed because it is an indicator of postpartum depression [21].

The prevalence is lower than the prevalence of postnatal depression (18.6%), anxiety (13.1%), and stress (8.7%) found in a representative sample of Qatari women who attended a primary health care postpartum consultation within 6 months after delivery [22]. Other studies in Australia [23] and Zimbabwe [24] showed similarly high levels of postnatal depression (17.4% and 16%, respectively). The WHO and the United Nations Population Fund reported 10%–15% postnatal depression in industrialized countries [25]. However, in our study, we did not specifically look for this type of health outcome. Certainly, our findings suggest the need to further explore postpartum mental disorders using appropriate tools. We hope that, given that mental distress ranked first among all self-reported postpartum health problems, postpartum depression in Morocco will receive more attention and bring postnatal depression ‘out of the shadows’ [26]. The second most common complaint was symptoms of genital infections (8%). It should be noted that 91% of women did not seek care.

The reasons given for not seeking care were distance from home to the maternity hospital, no pain, a bad experience during childbirth (for those who delivered at the regional maternity hospital), and fear of having a serious illness. These factors should be further studied so that interventions can be defined and implemented to increase the number of women who seek medical care for postpartum health problems. Subnotification of pathologies compared with observed measurements was demonstrated to be influenced first by age, education, and socioeconomic level and, second, by asymptomatic or vague signs of genital infection [27].

Our study also shows that a high proportion of women (60%) were given a diagnosis of a postnatal problem 6 to 8 weeks after delivery. Similar levels of prevalence, or higher (72%), were reported in other studies [16,28]. As with the prevalence of self-reported complaints, these medical diagnoses are a mixture of severe and benign. Only 12 women (1%) who attended the 42-day postpartum consultation had a severe condition such as an infected perineal tear, infected caesarean section or episiotomy wound, third-degree uterine prolapse, cervical cancer, or severe anemia. These women, who apparently were not aware of the severity of their problem, were immediately referred to the university hospital.

Of interest, only 85% of diagnoses were aligned with women’s self-reported complaints. Two health problems, in particular, account for this difference: psychological complaints and diagnoses of anemia. The physician recognized psychological complaints in only 5% of women, only half of the women who voiced such a complaint. Informal discussions with physicians indicate that they often did not take this type of complaint seriously, implying that anxiety, stress, tiredness, or crying were ‘normal’ in postpartum women and not important enough to be diagnosed or treated. Moreover, in Moroccan physicians’ training, significantly more attention is devoted to physical health than to mental health. Anemia was diagnosed in 19% of women, but only a small proportion of them complained of symptoms that could be attributed to anemia, and none were from women with hemoglobin >10 g Hb/dL. Physicians were able to diagnose anemia in this instance because the women were invited to take the laboratory test for free. Under normal circumstances, women would have to pay to get the test, and most would be unable to pay. The question remains as to whether a more specific test (eg, ferritin to measure iron stock) is required before treating anemia with iron.

Estimates of self-assessed morbidity prevalence are generally more specific than sensitive [29]. Underestimation or overestimation by women tends to be influenced by age, level of education, and specificity of clinical symptoms [27]. In industrialized countries, greater value is given to women’s self-reports of complaints, and responding to these complaints is considered more important than measuring the prevalence of true postpartum morbidity [2,30,31].

Today in Morocco, the proportion of women who attend a postnatal consultation at 6 weeks is 22% [14]. We believe that our study has brought clear advocacy arguments to increase this percentage. In our study, we were able to reach 80% of potential subjects by recruiting women at delivery and inviting them to return 6 weeks later for a health consultation by providing a voucher to get a free blood test and calling the small proportion of women who did not show up for their appointment.

### Limitations

This study had several limitations. First, although reminders were sent and women were called by phone, 20% did not attend the postnatal consultation, either because they did not want to or they had moved. This relatively high loss to follow up after delivery may have biased our results. We recorded the sociodemographic characteristics of these women and compared them with those who attended the consultation. Women with a lower education level, lower socioeconomic status, primiparae, delivered at home or at the regional hospital, and who did not have a complication were less likely to attend the postnatal consultation. Second, psychological complaints were recorded only when women brought them up themselves, but we did not specifically ask questions about mental distress to screen for postpartum depression as part of the standard interview. Therefore, it is likely that our findings are an underestimate of the true burden of this problem. Third, of a total of 43 single mothers, only 23 were recruited, and 20 of them did not agree to participate. This was likely a missed opportunity to investigate a particularly vulnerable group of women.

### Policy implications

A strategy to reduce morbidity, along within increased political backing and resources, are clearly needed to expand the scope of current programs to address the full range of maternal morbidities. Preventive strategies are focused on the prenatal and on the delivery period; however, our study highlighted the importance of addressing health problems experienced during the postpartum period. Integrated mechanisms to prevent or reduce the burden of the problems revealed by this study might consolidate and encourage the use of maternal health services. We must implement specific and innovative postpartum interventions and consider women’s mental health. These interventions must be included as part of the continuum of care, from the prenatal period through the postpartum phase.

Our findings provided new information about postpartum problems experienced by women in the Marrakech area, where mental health disorders in postpartum women were often not addressed by their physician. We need to sensitize and train medical doctors to pay more attention to women’s complaints (mental distress in particular) and appropriately manage them. In addition, the use of health services during pregnancy, at delivery, and during the postpartum period, is essential to avoid most obstetric complications, provided that they are quickly treated when they occur. Women’s use of mental health services is equally important in preventing reproductive morbidity. Their use of these services will depend on the woman’s perception of need, as well as on the availability, accessibility and, especially, the quality of these services.

## Conclusions

This analysis of the magnitude of postpartum morbidity revealed a possible hidden burden among Moroccan women. The comparison of women’s perceptions with their medical diagnoses highlighted the need to train physicians to listen to their patients and recognize the signs of psychological distress. Indeed, postpartum consultation should take into account women’s complaints, which are often not acknowledged or expressed. This study showed the importance of late (6 weeks) postpartum consultations. We were able to reach 80% of women in this study because women were invited at hospital discharge after delivery to attend the consultation and were actively followed by phone to remind them of their appointments. Making postpartum maternal morbidity a priority on the national agenda will help the fight against obstetric complications, which is cri tical to the wellbeing of women and their infants.

## Competing interests

The authors declare that they have no competing interests.

## Authors’ contributions

BA, AM, and VDB designed the study. BA conducted the study under the supervision of VDB. BA, VDB, and DD analyzed the data. BA wrote the initial draft of the manuscript. VDB, BD, and DD reviewed and critically revised the manuscript. All authors read and approved the final manuscript.

## Pre-publication history

The pre-publication history for this paper can be accessed here:

http://www.biomedcentral.com/1471-2393/13/225/prepub

## Supplementary Material

Additional file 1 Definitions and diagnostic tools selected by physicians who performed the postpartum consultations. Click here for file
