# Supplementary material for: Maternal postpartum morbidity in Marrakech: what women feel what doctors diagnose?
Source: BMC Pregnancy Childbirth. 2013 Dec 5;13:225. doi: 10.1186/1471-2393-13-225 (PMC3878998; doi:10.1186/1471-2393-13-225)
Supplement: Additional file 1 — Definitions and diagnostic tools selected by physicians who performed the postpartum consultations. [file 1471-2393-13-225-S1.docx]

**Additional file 1: Definitions and diagnostic tools morbidities selected by physicians who have made the consultation postpartum women**

| **Maternal morbidity/**  **disability** | **Definition** | **Clinical examination and specific biomedical tests** |
| --- | --- | --- |
| Prolapse | *Uterine prolapse*: Uterus descending into the vaginal canal with cervix as leading edge.  *Utero-vaginal prolapse*: Uterine prolapse pulling down the vagina.  *Vaginal prolapse*: One or more region of vaginal wall protrude into the vaginal canal.**[17]** | Interview with the woman and clinical examination |
| Anal incontinence | Involuntary loss of stool for a period of three months |  |
| Urinary tract infection (UTI) | Burning urination **[17]** |  |
| Anemia | Severe anemia—Hemoglobin level <7 g/dL  Moderate anemia = Hemoglobin level ≥7-9g/dL  Mild anemia = Hemoglobin level > 9-≤11g/dL**[17]** | Hemoglobinometry |
| Breast problems | Breast infection/Mastitis—pain, localized mass, tenderness, redness of breast Abscess- Breast mass with fluctuation. **[18]** | Visual inspection and manual palpation of the breast |
| Hypertension | Systolic blood pressure ≥140 mmHg or diastolic Blood pressure ≥90 mmHg**[15]** | Sphygmomanometer |
| External Hemorrhoids | Varicosities of the veins that occur outsidethe anal verge (the distal end of the anal canal),sometimes extruded as thrombosed structurestrangulated by anal sphincter**[17]** | Clinical examination |
| Urinary incontinence | Involuntary loss of urine weekly or more often measured by Cough Reflex Test**[19]** | Cough Reflex Test: Women wereasked to take 500 ml of water 45 minutes prior to pelvic examination and advised to walk around. During visual inspection, womenwere asked to cough strongly to see if urine was released |
| Perineal tear | Injury to the perineum involving perineal muscles with or without anal sphincter involvement, we have 4 degrees.**[17]** | Clinical examination |
| Depression post-partum | Two or more of the following symptoms in the same period of 2 weeks representing a change from normal:  •Guilt inappropriate or negative  •Feeling negative towards yourself.  •Crying easily.  •Decreased interest or pleasure.  •Feeling tired, agitated all the time.  •Disturbed sleep  •Diminished ability to think or concentrate  •Marked loss of appetite **[15]** | Interview with womanto identify signs of depression |
| Genital tract Infections | Vaginal discharge associated with itching or irritation, vaginal discharge or color with or without foul odor | Interview with the woman and clinical examination |
| Tract infection lower urinary | Pain or burning when urinating and frequent urination.**[17]** | Interview with the woman and lab examination |
| Vaginal Bleeding | Bleeding during the postpartum period which required the woman to change the fabric used to contain blood every hour or more often | Interview with the woman and clinical examination |
